# Supplementary material for: A Preliminary Study on Cross-Reactivity of Heat-Treated Quail and Hen’s Egg White Proteins in Young Children
Source: Nutrients. 2021 Jun 24;13(7):2172. doi: 10.3390/nu13072172 (PMC8308246; doi:10.3390/nu13072172)
Supplement: Supplementary file 1 [file nutrients-13-02172-s001.zip › nutrients-1270959-supplementary.pdf]

Supplementary Material

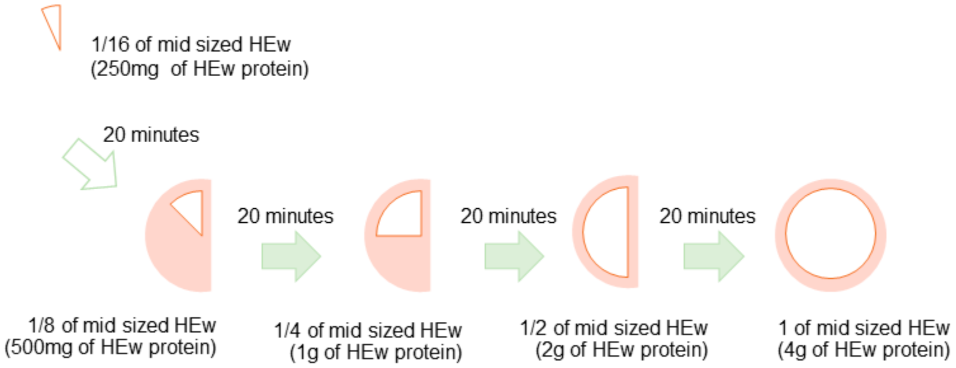

Figure S1. – Oral food challange protocol using hen's egg white. Protein data were reported by the National. Institute of Agricultural Sciences of Korea (2020) (http://koreanfood.rda.go.kr/). HEw, hen's egg white.

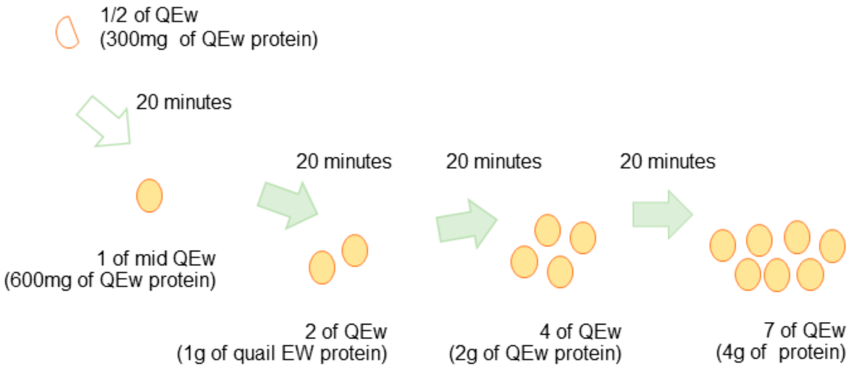

Figure S2. – Oral food challange protocol using quail egg white. Protein data were reported by the National. Institute of Agricultural Sciences of Korea (2020) (http://koreanfood.rda.go.kr/). QEw, hen's quail egg white.
